# The Standard Dictionary of Funk & Wagnalls

**Published:** 1895-11

**Authors:** 


The Standard Dictionary of Funk & Wagnalls.

The *St. James's Budget*, the weekly edition of the *St. James's Gazette*, London, one of the most conservative and authoritative of English journals, in the issue of July 27th, ends its review of the Funk & Wagnalls *Standard Dictionary*, as follows:

" Nothing can be more complete than this, nothing more exhaustive. * * * The excellencies of this book are so bewildering that whatever might be said of them there would be as much remaining to be said. To say that it is perfect in form and scope is not extravagance in praise, and to say that it is the most valuable Dictionary of the English language is but to repeat the obvious. The *Standard Dictionary* should be the pride of literary America, as it is the admiration of literary England."

The English critics on all sides show no hesitation in placing this new American Dictionary above all similar British works. The *Leeds Mercury* in a review just published says:

"We have no hesitation in stating that the Funk & Wagnalls *Standard Dictionary* is the best and most complete Dictionary of the English Language now in existence."

John Bull can be generously fair when he tries to be, but he cannot help but show just a little natural sensitiveness at being constrained to look elsewhere for a Dictionary of his own language: "Strange," the *London Literary World* exclaims in speaking of the *Standard Dictionary,* "that the Queen's English should find its chief autocrats in the country of the President."

As English comment is just now particularly severe on many things American, it is gratifying so see how unqualified is its continued praise of the *Standard Dictionary.* This is all the more remarkable in view of the natural sensitiveness on the part of England at being obliged to look elsewhere for a Dictionary of its own language.

